# Lateral hypothalamic orexin and melanin-concentrating hormone neurons provide direct input to gonadotropin-releasing hormone neurons in the human

**DOI:** 10.3389/fncel.2015.00348

**Published:** 2015-09-04

**Authors:** Katalin Skrapits, Vivien Kanti, Zsófia Savanyú, Csilla Maurnyi, Ottó Szenci, András Horváth, Beáta Á. Borsay, László Herczeg, Zsolt Liposits, Erik Hrabovszky

**Affiliations:** ^1^Laboratory of Endocrine Neurobiology, Institute of Experimental Medicine, Hungarian Academy of SciencesBudapest, Hungary; ^2^Department and Clinic for Production Animals, Faculty of Veterinary Science, Szent István UniversityÜllő, Hungary; ^3^MTA-SZIE Large Animal Clinical Research Group, Dóra majorÜllő, Hungary; ^4^Department of Forensic Medicine, Faculty of Medicine of the University of DebrecenDebrecen, Hungary; ^5^Department of Neuroscience, Faculty of Information Technology and Bionics, Pázmány Péter Catholic UniversityBudapest, Hungary

**Keywords:** hypothalamus, human, immunohistochemistry, melanin-concentrating hormone, orexin

## Abstract

Hypophysiotropic projections of gonadotropin-releasing hormone (GnRH)-synthesizing neurons form the final common output way of the hypothalamus in the neuroendocrine control of reproduction. Several peptidergic neuronal systems of the medial hypothalamus innervate human GnRH cells and mediate crucially important hormonal and metabolic signals to the reproductive axis, whereas much less is known about the contribution of the lateral hypothalamic area to the afferent control of human GnRH neurons. Orexin (ORX)- and melanin-concentrating hormone (MCH)-synthesizing neurons of this region have been implicated in diverse behavioral and autonomic processes, including sleep and wakefulness, feeding and other functions. In the present immunohistochemical study, we addressed the anatomical connectivity of these neurons to human GnRH cells in *post-mortem* hypothalamic samples obtained from autopsies. We found that 38.9 ± 10.3% and 17.7 ± 3.3% of GnRH-immunoreactive (IR) perikarya in the infundibular nucleus of human male subjects received ORX-IR and MCH-IR contacts, respectively. On average, each 1 mm segment of GnRH dendrites received 7.3 ± 1.1 ORX-IR and 3.7 ± 0.5 MCH-IR axo-dendritic appositions. Overall, the axo-dendritic contacts dominated over the axo-somatic contacts and represented 80.5 ± 6.4% of ORX-IR and 76.7 ± 4.6% of MCH-IR inputs to GnRH cells. Based on functional evidence from studies of laboratory animals, the direct axo-somatic and axo-dendritic input from ORX and MCH neurons to the human GnRH neuronal system may convey critical metabolic and other homeostatic signals to the reproducive axis. In this study, we also report the generation and characterization of new antibodies for immunohistochemical detection of GnRH neurons in histological sections.

## Introduction

In all mammals including the human, neurons synthesizing type-I gonadotropin-releasing hormone (GnRH) form the final common output way from the hypothalamus in the neuroendocrine control of reproduction. Failure of these cells to finish their prenatal migration from the olfactory placode to the forebrain results in hypogonadotropic hypogonadism characterized by the absence of puberty and reproductive capacity (Schwanzel-Fukuda and Pfaff, 1989). The decapeptide GnRH is secreted into the hypophysial portal circulation in an episodic manner (Carmel et al., 1976; Clarke and Cummins, 1982) to regulate the synthesis and secretion of the two gonadotroph hormones follicle-stimulating hormone and luteinizing hormone (LH) by the anterior pituitary (Belchetz et al., 1978).

Hormonal and metabolic signals influencing the reproductive axis at the hypothalamic level either regulate GnRH cells directly or act on the neuronal circuitry upstream from the GnRH neuron. Anatomical information about the neuronal systems regulating human GnRH neurons via afferent connections have been summarized in recent review articles (Dudás and Merchenthaler, 2006; Hrabovszky and Liposits, 2013). Such afferents originate from hypothalamic as well as extrahypothalamic sources and use monoamines, amino acids and a variety of neuropeptides for neuronal transmission (Dudás and Merchenthaler, 2006; Hrabovszky and Liposits, 2013).

Most peptide neurotransmitters identified within neuronal afferents to human GnRH neurons are synthesized in distinct cell populations of the medial hypothalamus (Hrabovszky and Liposits, 2013) and include neuropeptide Y (Dudás et al., 2000), substance P (Dudás and Merchenthaler, 2002a; Hrabovszky et al., 2013), galanin (Dudás and Merchenthaler, 2004a), corticotropin-releasing hormone (Dudás and Merchenthaler, 2002b), kisspeptin (Hrabovszky et al., 2010, 2011, 2012b; Molnár et al., 2012), neurokinin B (Hrabovszky et al., 2011, 2012b; Molnár et al., 2012), endorphins (Dudás and Merchenthaler, 2004b), enkephalins (Dudás and Merchenthaler, 2003), dynorphins (Dahl et al., 2009), RF-amide related peptide/gonadotropin-inhibiting hormone (Ubuka et al., 2009) and cocaine- and amphetamine-regulated transcript (Skrapits et al., 2014).

The lateral hypothalamic area (LHA) is crucially involved in the control of diverse behavioral and autonomic processes, including respiration (Burdakov et al., 2013), sleep and wakefulness (Konadhode et al., 2015), feeding and drinking (Kunii et al., 1999; Watts et al., 2007; Burdakov et al., 2013) and reproduction (Pu et al., 1998; Wu et al., 2009). This site hosts orexin (ORX)- and melanin-concentrating hormone (MCH)-synthesizing neurons, among other cell-types. Both ORX and MCH neurons send wide projections in different species. Of particular interest, these fibers abundantly innervate the preoptic region, where GnRH-immunoreactive (IR) neuronal cell bodies reside, and the mediobasal hypothalamus, where the hypophysiotropic GnRH terminals are found (Bittencourt et al., 1992; Peyron et al., 1998; Date et al., 1999; Nambu et al., 1999). The ORX-containing fibers in rats (Campbell et al., 2003) and sheep (Iqbal et al., 2001) and the MCH-IR fibers in male mice (Ward et al., 2009) and female rats (Williamson-Hughes et al., 2005) were reported to provide direct inputs to GnRH neurons. Electrophysiological studies on GnRH-GFP mice have also shown that GnRH neurons possess functional receptors for these neuropeptides (Campbell et al., 2003; Wu et al., 2009; Gaskins and Moenter, 2012). These peptidergic inputs from the LHA to GnRH neurons may significantly contribute to the functional link between the regulation of food intake and reproduction (López et al., 2010). The issue of whether peptidergic cell populations of the LHA play any role in the afferent control of human GnRH neurons requires clarification.

In the present study, we investigated the putative projections of lateral hypothalamic ORX and MCH neurons to preoptic/medial hypothalamic GnRH cells in humans by performing immunohistochemical studies of *post-mortem* hypothalamic samples obtained at autopsies. Investigation of these anatomical links with dual-labeling immunohistochemistry has been supplemented with quantitative analyses to determine: (i) the percentages of GnRH-IR perikarya receiving ORX-IR and MCH-IR contacts; (ii) the mean incidences of ORX-IR and MCH-IR afferent contacts on GnRH-IR cell bodies; (iii) the average number of axo-dendritic contacts per 1 mm segment of GnRH dendrites; and (iv) the relative incidences of axo-somatic *vs.* axo-dendritic contacts on GnRH-IR neuronal elements. We also report the generation and characterization of several new antibodies capable of recognizing GnRH neurons in immunohistochemical assays.

## Materials and Methods

### Human Subjects

Human hypothalamic tissue samples from five male (ages 21–78 years) and two female (ages 56 and 59 years) subjects who died from sudden causes of death were obtained at autopsy from the Forensic Medicine Department of the University of Debrecen. Permission was obtained from the Regional Committee of Science and Research Ethics (DEOEC RKEB/IKEB: 3183-2010). The history of patients and autopsy diagnoses did not indicate previous neurological and endocrine disorders.

### Tissue Preparation for Immunohistochemistry

Autopsies were carried out within 48 h after death. Hypothalamic tissue blocks were dissected out, rinsed with running tap water and then, immersion-fixed in 4% formaldehyde in 0.1 M phosphate buffered saline (PBS; pH 7.4) for 14 days. Then, the blocks were cut in half in the midsagittal plane, trimmed, infiltrated with 20% sucrose (5 days, 4°C) and cryo-sectioned coronally at 30 μm with a Leica SM 2000R freezing microtome (Leica Microsystems, Nussloch GmbH, Germany), as described earlier (Hrabovszky et al., 2010, 2011, 2012b, 2013; Molnár et al., 2012; Skrapits et al., 2014). The sections were stored permanently in anti-freeze solution (30% ethylene glycol; 25% glycerol; 0.05 M phosphate buffer; pH 7.4) at −20°C.

### Animal Tissues Used to Test the Performance of Newly-Developed GnRH Antibodies

Adult male CD1 mice (*N* = 2) and Wistar rats (*N* = 2) were used from local breeding colonies of the Medical Gene Technology Unit of the Institute of Experimental Medicine. They were deeply anesthetized with a cocktail of ketamine (25 mg/kg), xylavet (5 mg/kg) and pipolphen (2.5 mg/kg) in saline and sacrificed by transcardiac perfusion with 10 ml of a 0.1 M PBS, followed by 4% paraformaldehyde in 0.1 M PBS. The brains were removed, postfixed for 1 h in the same fixative, infiltrated with 20% sucrose overnight and then, snap-frozen on dry-ice. Preoptic/hypothalamic blocks were dissected and 30-μm-thick coronal sections were prepared on a freezing microtome (Leica). All experiments were carried out in accordance with the Council Directive of 24 November 1986 of the European Communities (86/609/EEC) and approved by the Animal Welfare Committee of the Institute of Experimental Medicine (No. A5769-01).

### Tissue Pretreatments for Immunohistochemistry

Prior to immunohistochemistry, the sections were rinsed in PBS and pretreated with a mixture of 0.5% H_2_O_2_ and 0.5% Triton X-100 for 30 min. In case of human tissues, this was followed by antigen retrieval using 0.1 M citrate buffer (pH 6.0) at 80°C for 30 min.

### Experiment 1: Generation and Characterization of GnRH and hGAP1 Antibodies in Different Host Species

A previously characterized reference GnRH antiserum (EH#1018) has been generated in guinea pig against type-1 (mammalian) GnRH conjugated to bovine thyroglobulin with 1-ethyl-3-(3-dimethylaminopropyl) carbodiimide in 100 mM MES buffer (pH 4.7; Hrabovszky et al., 2011). Here we used the same antigen preparation to raise GnRH antibodies in one rat and two sheep. In addition, another antigen construct was used to raise polyclonal antibodies in a mouse against a 14-amino acid segment of the human GnRH-associated peptide 1 (hGAP1).

#### Rat GnRH Antibodies (EH#1044)

One rat (#1044) was immunized intraperitoneally (i.p.) with 100 μg antigen/injection in 300 μl volume. The ratio of the aqueous phase and the adjuvant was always 1:9. The first injection was carried out with Freund’s complete adjuvant. To induce ascites production, 500 μl Pristane (Sigma, St. Louis, MO, USA) was injected i.p. to each rat on day 6. Subsequent boosts were given i.p. with Freund’s incomplete adjuvant at 3-week intervals. Ascites (EH#1044) was collected 8 days after booster injections and tested with immunohistochemistry as well as immunofluorescence at various dilutions on tissue sections of the mouse medial preoptic area (containing GnRH cell bodies), organum vasculosum of the lamina terminalis (containing many GnRH cell bodies and processes) and eminentia mediana (containing the hypophysiotropic GnRH axon terminals).

#### Sheep GnRH Antibodies (EH#2000 and EH#2001)

To generate GnRH antibodies in sheep, two one-year-old gimmers were immunized subcutaneously with 1000 μg (sheep #2001) and 2000 μg (sheep #2000) antigen/injection, respectively. The ratio of the aqueous phase and the adjuvant was 1:1 in 2 ml volumes. Subsequent boosts were given monthly. Eight days after each booster injection, serum samples were withdrawn from the jugular vein and tested with immunohistochemistry on mouse and human preoptic/hypothalamic tissue sections. The sheep were exsanguinated after 3 months when appropriate serum titers were achieved. The blood was allowed to clot and the sera of the two sheep (EH#2000 and EH#2001) were collected by centrifugation. Sodium azide was added at 0.1% and then, the antiserum aliquots were frozen and transferred to −20°C for long-term storage.

#### Mouse Antibodies (EH#1001) Against hGAP1

A 14-amino acid synthetic peptide (FECTTHQPRSPLRD) corresponding to amino acids 61–74 of the human proGnRH1 and to amino acids 24–37 of hGAP1 (accession: P01148) was used as hapten. The homologous mouse and rat sequences exhibit two amino acid replacements (V for T at position 65 and W for Q at position 67). Four mg peptide was conjugated to 25 mg bovine serum albumin with 12 mg 1-ethyl-3-(3-dimethylaminopropyl) carbodiimide in 4 ml 100 mM MES buffer (pH 4.7). Unconjugated peptides were removed with dialysis against 0.1 M PBS (pH 7.6; 3 × 8 h; Hrabovszky et al., 2011). The conjugate was used to immunize two mice i.p. with the same doses and schedule as described above for the generation of GnRH antibodies in rats. Ascites (EH#1001) was collected 8 days after boosts and tested on human and rat preoptic/hypothalamic sections.

#### Characterization of the New GnRH and hGAP1 Antibodies

The new GnRH and hGAP1 antibodies were tested for labeling specificity using several approaches. These included use of the biotinylated secondary antibody (donkey biotin-SP-anti-rat, sheep or mouse IgG; Jackson ImmunoResearch Laboratories, West Grove, PA, USA; 1:500)-ABC method (ABC Elite reagent; Vector, Burlingame, CA, USA; 1:1000), with nickel-diaminobenzidine in the peroxidase developer. In addition, fluorochrome-conjugated secondary antibodies (Jackson ImmunoResearch Laboratories) were used for immunofluorescent experiments. Negative controls to demonstrate the specificity of immunolabeling included the omission of the primary antibodies from the immunohistochemical protocol or the overnight preabsorption of the antibody working solutions with 1 μg/ml of the synthetic peptides used for conjugation. As a positive control, the new antibodies were used in parallel assays on neighboring test sections of mice or humans, and the labeling patterns were compared to those obtained with the reference guinea pig GnRH antiserum (EH#1018; Hrabovszky et al., 2011). Immunofluorescent dual-labeling experiments also assessed the co-labeling of GnRH perikarya and fibers with the different antibodies using secondary antibodies conjugated to distinct fluorochromes (Jackson ImmunoResearch Laboratories; FITC, 1:250; Cy3, 1:1000). Section mounting, coverslipping and the microscopic analyses following peroxidase- and fluorescent immunolabeling were carried out as described below for experiments 2 and 3, respectively.

### Experiment 2: Dual-Immunoperoxidase Detection of ORX-IR and MCH-IR Inputs to GnRH-Synthesizing Neurons

First, every 24th section from each individual was incubated in a goat polyclonal ORX B antiserum (sc-8071; C-19, 1:50,000; Santa Cruz Biotech Inc., Santa Cruz, CA, USA; Bullmann et al., 2010) for 48 h at 4°C. A second set of sections was reacted similarly with a goat polyclonal proMCH antiserum (sc-14509; C-20, 1:4000; Santa Cruz, CA, USA; Whiddon and Palmiter, 2013). The primary antibodies were reacted with biotinylated secondary antibodies (donkey biotin-SP-anti-goat IgG; Jackson ImmunoResearch Laboratories; 1:500) and the ABC Elite reagent (Vector; 1:1000) for 60 min each. The peroxidase signal was visualized with nickel-intensified diaminobenzidine chromogen and then, post-intensified with silver-gold (Liposits et al., 1984).

Following the detection of ORX-IR or MCH-IR neurons, the sections were incubated overnight in polyclonal GnRH antibodies raised in one of three different host species (guinea pig, 1:30,000, rat, 1:30,000 and sheep, 1:30,000), or alternatively, in the newly generated hGAP1 (1: 30,000) antibodies. The primary antibodies were reacted with biotinylated secondary antibodies (Jackson ImmunoResearch Laboratories; 1:500; 1 h) and the ABC Elite reagent (Vector; 1:1000; 1 h) and then, the peroxidase signal was visualized with brown diaminobenzidine chromogen. The sections were mounted on microscope slides from Elvanol, air-dried, dehydrated with 95% (5 min), followed by 100% (2 × 5 min) ethanol, cleared with xylene (2 × 5 min) and coverslipped with DPX mounting medium (Sigma). Representative light microscopic images were prepared with an AxioCam MRc 5 digital camera mounted on a Zeiss AxioImager M1 microscope and using the AxioVision 4.6 software (Carl Zeiss, Göttingen, Germany).

### Experiment 3: Confocal Analysis of ORX-IR and MCH-IR Inputs to GnRH-Synthesizing Neurons

These studies were performed to confirm the presence of direct contacts between the ORX-IR and MCH-IR axons and the GnRH-IR perikarya and/or processes at the confocal level. Two sets of sections were incubated in the sc-8071 ORX B (1:2000) and the sc-14509 proMCH (1:500) antibodies, respectively, for 48 h at 4°C. The goat primary antibodies were reacted with Cy3-conjugated donkey anti-goat IgG (Jackson ImmunoResearch Laboratories; 1:1000; 5 h). To detect GnRH, the guinea pig GnRH antibodies (EH#1018; 1:3000) were used for 48 h at 4°C, followed by FITC-conjugated donkey anti-guinea pig antibodies (Jackson ImmunoResearch Laboratories; 1:250; 5 h). The immunofluorescent specimens were mounted on glass slides from 0.1 M Tris-HCl buffer (pH 7.6) and coverslipped with the aqueous mounting medium Mowiol. The dual-labeled fluorescent sections were analyzed using a Zeiss LSM 780 confocal microscope with a 20x/0.8 NA objective and the Zen software (Carl Zeiss). To illustrate the results, confocal *z*-stacks were used which also showed the orthogonal side-views of ORX-IR and MCH-IR appositions.

### Experiment 4: Quantitative Analysis of ORX-IR and MCH-IR Inputs to the Somatic and Dendritic Compartments of GnRH Neurons in the Infundibulum

Two series of sections containing the infundibular nucleus (INF) were immunostained with the dual-immunoperoxidase method to detect ORX-IR and MCH-IR inputs to GnRH neurons, as described in experiment 2. GnRH neurons were visualized using the guinea pig antiserum (EH#1018) and several quantitative aspects of their ORX-IR and MCH-IR innervation were studied in histological specimens of the five male subjects. The analysis of neuronal appositions was carried out using a 63× oil-immersion objective. A contact was defined using stringent criteria that were applied consistently. The axon and the GnRH profile had to be in the same focus plane without any visible intervening gap and instances of partial overlap were not considered (Hrabovszky et al., 2011, 2012a). First, the percent ratios of GnRH-IR perikarya that received at least one afferent contact and the average number of axo-somatic contacts were determined for both the ORX/GnRH and the MCH/GnRH dual-labeling experiments. The analyses included 63 and 64 GnRH neurons, respectively. In the absence of a sharp transition between the somatic and dendritic compartments of the typically elongated GnRH neurons, ORX-IR and MCH-IR inputs identified on GnRH-IR profiles ticker than 3 μm were considered axo-somatic. Next, the sections were analyzed to determine the average number of afferent contacts per mm of GnRH dendrite. A total length of 28.385 mm GnRH dendrites found in eight sections of five male individuals were analyzed for ORX-IR inputs and 13.894 mm GnRH dendrites in 10 sections of five male individuals for MCH-IR inputs. A final analysis determined the percentages of the encountered ORX/GnRH (*N* = 219) and MCH/GnRH (*N* = 67) appositions that targeted the dendritic *vs.* the somatic compartment of GnRH neurons. Beaded GnRH-IR processes which had axonal rather than dendritic appearances as well as GnRH processes within or close to the infundibular stalk were excluded from this analysis.

## Results

### Experiment 1: Generation and Characterization of GnRH and hGAP1 Antibodies in Different Species

**Figure 1:**
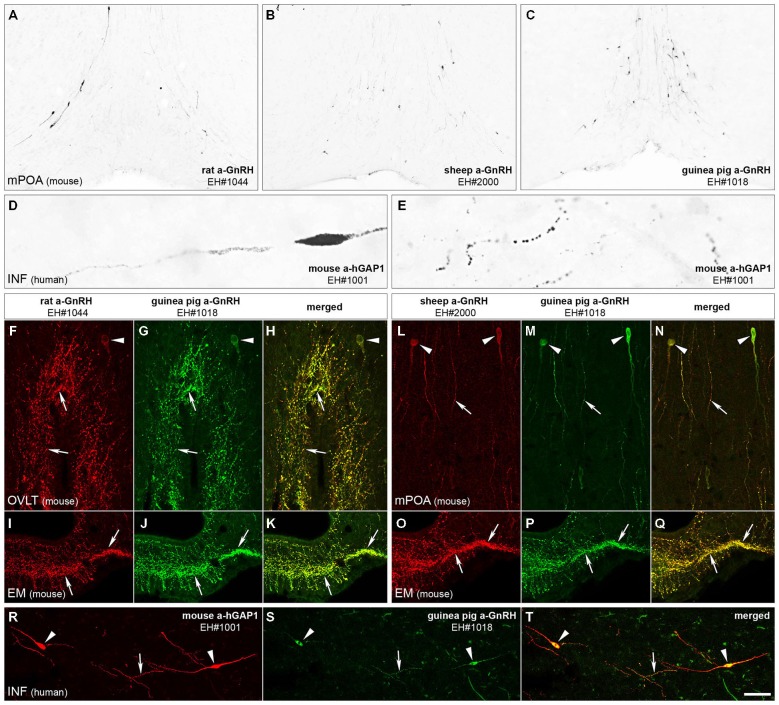
**Immunohistochemical characterization of GnRH and hGAP1 antibodies**. The validation of immunohistochemical labeling with the newly generated rat GnRH (EH#1044; **A,F,I**), sheep GnRH (EH#2000; **B,L,O**) and mouse hGAP1 (EH#1001; **D,E,R**) antibodies used a reference guinea pig GnRH antiserum (EH#1018; **C,G,J,M,P,S**) as positive control in light **(A–E)** and confocal **(F–T)** microscopic specimens. Light micrographs of nickel-diaminobenzidine-stained sections illustrate the distribution of GnRH-IR cell bodies in the medial preoptic area (mPOA) of the mouse using the new rat (EH#1044; 1:200,000; **A**) and sheep (EH#2000; 1:200,000; **B**) GnRH antibodies and the guinea pig (positive control) antiserum (EH#1018; 1:200,000; **C**). Note that all three antibodies reveal typical distribution patterns of preoptic GnRH neurons. Antibodies #1001 raised in mouse ascites fluid against the hGAP1 can label intensely the scattered GnRH-IR cell bodies **(D)** and their processes **(E)** in the human hypothalamus, including its infundibular nucleus (INF). A series of dual-immunofluorescent experiments confirms that the new antibodies recognize the same perikarya (arrowheads) and axons (arrows) as the reference guinea pig GnRH antiserum. Panels **(F–H)** illustrate structures dual-labeled with the rat (1:5000)/guinea pig (1:5000) antiserum combination around the organum vasculosum of the lamina terminalis (OVLT) and also in the eminentia mediana (EM; **I–K**) where hypophysiotropic GnRH axons project. Panels **(L–Q)** reveal the dual-labeling of GnRH-IR structures in the mPOA **(L–N)** and the EM **(O–Q)** using the sheep (1:10,000)/guinea pig (1:5000) antiserum combination. Similarly, GnRH neurons of the human can be dual-labeled with the combined use of the mouse hGAP1 (1:5000) and the guinea pig GnRH (1:5000) antibodies **(R–T)**. Scale bars = 100 μm in **(A–C)**, 16 μm in **(D,E)**, 25 μm in **(F–T)**.

The immunohistochemical detection of GnRH with the new rat (EH#1044; Figure 1A) and sheep (EH#2000 and EH#2001; Figure 1B) GnRH antibodies and the reference guinea pig GnRH antiserum (EH#1018; Figure 1C) revealed typical distribution patterns and morphology of GnRH-IR cell bodies in the medial preoptic area of mice. Intensely IR neurons were also visualized in the human, but not the rodent, hypothalmi using the newly-generated mouse antibodies against hGAP1 (EH#1001; Figures 1D,E). In negative control experiments, all labeling was eliminated if the primary antibodies were omitted from the procedure or preabsorbed overnight with 1 μg/ml of the peptide used for conjugation (not shown). In addition, positive control experiments using dual-immunofluorescent labeling with two different fluorochromes verified that the new rat (Figures 1F,I) and sheep (Figures 1L,O) GnRH antibodies and the mouse hGAP1 antibodies (Figure 1R) co-labeled the same cell bodies and fibers (Figures 1H,K,N,Q,T) that were recognized by the guinea pig reference GnRH antibodies EH#1018 (Hrabovszky et al., 2011; Figures 1G,J,M,P,S).

### Experiment 2: Dual-Immunoperoxidase Detection of ORX-IR and MCH-IR Inputs to GnRH-Synthesizing Neurons

**Figure 2:**
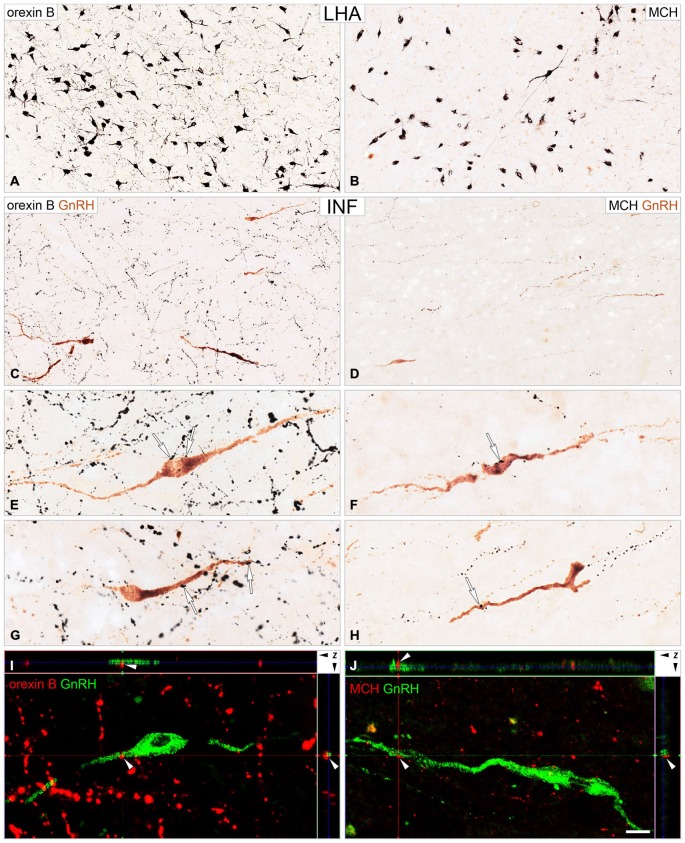
**Immunohistochemical detection of ORX-IR and MCH-IR inputs to GnRH neurons**. Light **(A–H)** and confocal **(I,J)** microscopic images demonstrate the distribution of ORX B (ORX)-IR and MCH-IR cell bodies (**A** and **B**, respectively) in the lateral hypothalamic area (LHA) and their efferents to GnRH neurons **(C–J)** of the infundibular nucleus (INF). Light micrographs illustrate ORX-IR and MCH-IR neuronal elements visualized with the black silver-gold intensified nickel-diaminobenzidine chromogen and GnRH neurons immunostained with brown diaminobenzidine. GnRH neurons in the INF are embedded into a dense ORX-IR plexus **(C)** and a somewhat less dense MCH-IR fiber network **(D)**. Arrows in high power photomicrographs **(E–H)** point to neuronal appositions between ORX-IR **(E,G)** and MCH-IR **(F,H)** axons and GnRH-IR perikarya **(E,F)** and dendrites **(G,H)**. Note the higher abundance of the ORX-IR *vs.* the MCH-IR inputs to GnRH cells, which was confirmed by the quantitative analysis of experiment 4 (Figure 3). Images from confocal *z*-stacks illustrate axo-dendritic appositions (white arrowheads) in the dual-immunofluorescent specimen **(I,J)**. Note the absence of gap between the juxtaposed profiles in the orthogonal side-views (*z* axis) of contacts. Scale bars = 50 μm in **(A,B)**, 25 μm in **(C,D)**, 16 μm in **(E–H)**, 12 μm in **(I,J)**.

Following the dual-immunoperoxidase labeling of hypothalamic sections, the silver-gold intensified nickel-diaminobenzidine chromogen visualized intensely-labeled ORX-IR (Figure 2A) and MCH-IR (Figure 2B) cell bodies in the LHA. The majority of GnRH cells labeled with brown diaminobenzidine chromogen were located in preoptic/medial hypothalamic regions; the same sites also received peptidergic axonal projections from the LHA (Figures 2C,D). High-power light microscopic analysis of the double-labeled preoptic and hypothalamic sections revealed ORX-IR (Figures 2E,G) and MCH-IR (Figures 2F,H) axonal appositions on subsets of the GnRH-IR perikarya (Figures 2E,F) and dendrites (Figures 2G,H).

### Experiment 3: Confocal Analysis of ORX-IR and MCH-IR Inputs to GnRH-Synthesizing Neurons

The existence of direct ORX-IR and MCH-IR inputs to GnRH neurons was analyzed and confirmed with confocal microscopy. The orthogonal side-views of *z*-stacks reconstructed from multiple optical slices ruled out that overlaps are mistakenly considered appositions (Figures 2I,J).

### Experiment 4: Quantitative Analysis of ORX-IR and MCH-IR Inputs to the Somatic and Dendritic Compartments of GnRH Neurons

**Figure 3:**
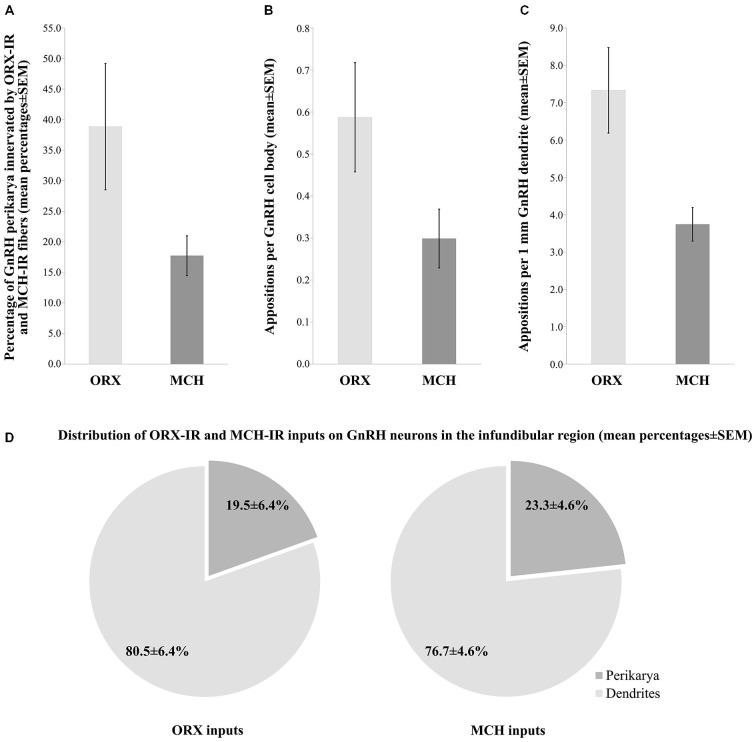
**Quantitative aspects of the ORX-IR and the MCH-IR innervation of GnRH neurons in the INF of human male subjects**. The percentages of GnRH-IR perikarya receiving ORX-IR and MCH-IR contacts **(A)**, the mean incidences of ORX-IR and MCH-IR afferent contacts on the perikarya of GnRH neurons **(B)**, the average number of axo-dendritic contacts per 1 mm GnRH dendrite **(C)** and the relative incidences of axo-dendritic *vs.* axo-somatic juxtapositions **(D)** were determined from five adult male human individuals. Note that GnRH neurons in these studies receive about twice as many inputs from ORX-IR neurons than from MCH-IR ones and both types of input preferentially target the dendritic compartment of GnRH neurons.

The number and heterogeneity of cases available for these studies did not allow us to address the issues of the putative sexual dimorphism and age-related changes in the innervation patterns. The quantitative light microscopic analysis of inputs to GnRH neurons of the INF was restricted to the dual-peroxidase stained specimen of male subjects and results were expressed as mean ± SEM of the five individuals. These studies have revealed ORX-IR contacts on 38.9 ± 10.3% and MCH-IR contacts on 17.7 ± 3.3% of GnRH-IR perikarya in the INF (Figure 3A). On average, GnRH-IR cell bodies received 0.6 ± 0.1 ORX-IR and 0.3 ± 0.07 MCH-IR inputs (Figure 3B). ORX-IR and MCH-IR appositions were more typically encountered on the processes of GnRH neurons. A quantitative analysis (from which axon-like varicose processes of GnRH cells were excluded) identified 7.3 ± 1.1 ORX-IR and 3.7 ± 0.5 MCH-IR axo-dendritic appositions on each 1 mm dendrite of GnRH neurons in the INF (Figure 3C). Overall, the axo-dendritic contacts represented the dominant form of inputs to GnRH cells, representing 80.5 ± 6.4% of the ORX-IR and 76.7 ± 4.6% of the MCH-IR inputs that were encountered (Figure 3D).

## Discussion

This immunohistochemical study provides neuroanatomical evidence for the direct innervation of human GnRH cells by ORX and MCH neurons of the lateral hypothalamus. We show that both types of inputs preferentially target the dendritic compartment of GnRH neurons. In addition, we describe the generation and characterization of new polyclonal rat and sheep GnRH and mouse hGAP1 antibodies suitable for the detection of GnRH-IR perikarya and processes.

### Generation of New Antibodies to Detect GnRH Neurons

In this study, we report the preparation and specificity testing of new antibodies allowing the immunohistochemical visualization of GnRH perikarya and their processes. The availability of such antibodies from optional host species will be particularly valuable in future immunofluorescent multiple-labeling experiments. Although antibodies against the mammalian form of GnRH decapeptide are readily available from commercial sources, many of such products require the colchicine pretreatment of the experimental rodents to achieve the sufficient visualization of the GnRH cell bodies. This technical limitation accounts for the wide use in publications of excellent private GnRH antisera; one of them (rabbit anti-GnRH; LR-1) has been made generously available for the wide scientific community by Dr. R. A. Benoit (Montréal, Canada). In a previous study, we have also used the LR-1 reference antibodies as a positive control to demonstrate the specificity of immunolabeling obtained with our two newly produced polyclonal guinea pig GnRH antisera EH#1018 and EH#1035 (Hrabovszky et al., 2011). In the present work, we report the generation of GnRH antibodies in two additional host species. At optimal dilutions, all of the new antibodies can recognize the cell bodies of GnRH neurons in immunohistochemical assays, in addition to visualizing their axonal and dendritic processes. The rat (EH#1044) and sheep (EH#2001 and EH#2000) GnRH antibodies as well as the mouse antibodies against hGAP1 (EH#1001) were equally suitable to visualize the perikarya of GnRH neurons in human sections. Test sections from rodents also exhibited excellent somatic labeling of GnRH neurons in the organum vasculosum of the lamina terminalis/preoptic regions using the rat and sheep antibodies against GnRH, but not the mouse antibodies directed against the hGAP1 sequence. Thus, the two amino acids substituted in the rodent GAP1 sequence appear to be important antigen determinants of the hGAP1 fragment we used to generate EH#1001. Our positive control studies established that the immunohistochemical/immunofluorescent images obtained using the new antibodies were similar to those provided by the use of our reference guinea pig GnRH antiserum (EH#1018). Indeed, in dual-immunofluorescent studies we confirmed that the new antibodies reacted specifically with the same neuronal elements as did the reference GnRH antiserum, if appropriate working dilutions were chosen for each.

### Role of ORX-ergic Neurons in the Afferent Regulation of Human GnRH Cells

ORX/hypocretin was first isolated from the rat hypothalamus in the late 1990’s. Its two isoforms ORX A and B are derived from the same 130-amino acid peptide precursor (Sakurai et al., 1998; de Lecea et al., 1998). ORXs are present in both the peripheral and central nervous systems. In several species including rat (Elias et al., 1998; de Lecea et al., 1998), mouse (Broberger et al., 1998; Elias et al., 1998) and human (Elias et al., 1998; Moore et al., 2001), ORX cells are distributed around the LHA. The projections of ORX neurons, in turn, can be found throughout the brain and the spinal cord (Broberger et al., 1998; Elias et al., 1998; Peyron et al., 1998; Date et al., 1999; van den Pol, 1999). In male and female rats, ORX-IR fibers abundantly innervate the preoptic region (Peyron et al., 1998; Date et al., 1999; Nambu et al., 1999) where the majority of GnRH cell bodies reside. Immunohistochemical studies revealed that 75–85% of GnRH perikarya in male and female rats (Campbell et al., 2003) and 30% in the ovine hypothalamus (Iqbal et al., 2001) receive direct appositions form ORX-IR axons. In the present light microscopic studies, we provided evidence that high percentages (~40%) of GnRH cell bodies in the INF of the human hypothalamus also receive direct ORX-ergic inputs. The innervation of GnRH neurons was confirmed at the confocal level. The isoforms of the ORX receptor (OX) mediating the putative effects of ORXs to human GnRH neurons have not been addressed in our study.

The ORX receptors (OX1 and OX2) use complex signal transduction processes in which the extracellular Ca^2+^ concentration plays a more critical role than the cytoplasmic Ca^2+^-stores. Right amount of extracellular calcium is essential for ORX binding, and Ca^2+^ influx also amplifies the responses mediated by OXs (Johansson et al., 2007; Kukkonen and Leonard, 2014). In rats, 85% of GnRH cells express the OX1 receptor form (Campbell et al., 2003) which is known to bind preferentially ORX A (Sakurai et al., 1998), whereas the OX2 receptor which binds the two ORX forms with similar affinity (Sakurai et al., 1998) has not been reported on GnRH cells so far. Multiple sites and mechanisms of action may complicate the interpretation of the variable reproductive effects of ORXs *in vivo*. ORX receptors are not only expressed in the brain but also in peripheral endocrine organs such as the pituitary, testis, epididymis, seminal vesicle, ovary and placenta (Jöhren et al., 2001; Karteris et al., 2004). In addition, ORXs also influence reproduction via acting at multiple hypothalamic sites. The LH surge of the estrogen plus progesterone-treated ovariectomized rats is inhibited by direct injections of ORX A into the mediobasal hypothalamus and the medial preoptic area and stimulated by injections placed around the organum vasculosum of the lamina terminalis (Small et al., 2003). ORX effects are influenced by the sex steroid levels of the experimental animal. Accordingly, central administration of ORXs either stimulate or suppress the pulsatile secretion of LH in ovariectomized rats, depending on the presence or the absence, respectively, of ovarian sex steroids (Pu et al., 1998; Tamura et al., 1999). ORX A causes more dramatic effects than ORX B, suggesting the involvement of the OX1 receptor form (Tamura et al., 1999). Of note, the steroid-dependent bimodal effects of ORXs are reminiscent to similar observations using another orexigenic peptide neuropeptide Y (McDonald, 1990). Results of electrophysiological studies indicate that the direct actions of ORXs on GnRH neurons are inhibitory. ORX A suppressed the firing frequency (both bursts and single spikes) of GnRH neurons via acting on OX1 receptor (Gaskins and Moenter, 2012). This suppression was different in the presence and absence of estrogen; in ovariectomized mice ORX A reduced GnRH activity in both the morning and evening, whereas GnRH activity was only reduced in the evening using ovariectomized mice treated with estradiol (Gaskins and Moenter, 2012).

One possible physiological relevance of the ORX system in fertility is linked to the adaptation of the reproductive axis to the metabolic state (Silveyra et al., 2007; López et al., 2010). It has also been proposed that ORXs may connect reproduction with the sleep-wake cycle (López et al., 2010). The pulsatile release of LH is diminished in ORX-deficient narcoleptic men (Kok et al., 2004), although they show normal reproductive capacity (Taheri et al., 2002), similarly to the prepro-ORX knockout mice (Chemelli et al., 1999). The direct axo-somatic and axo-dendritic input from ORX neurons to the human GnRH neuronal system may represent an important anatomical route in the communication channel whereby the LHA regulates the reproductive axis.

### Functional Significance of MCH in the Afferent Regulation of GnRH Neurons

MCH is a cyclic 19-amino-acid peptide originally isolated from the teleost pituitary (Kawauchi et al., 1983) and later detected also in the central nervous system of various species, including the human (Mouri et al., 1993). Neuronal cell bodies synthesizing MCH were localized similarly to the medial forebrain bundle-LHA, subzona incerta and the perifornical area in rodents (Skofitsch et al., 1985; Bittencourt et al., 1992), sheep (Tillet et al., 1996) and monkeys (Bittencourt et al., 1998). The dominant distribution of MCH-IR neurons in the LHA/perifornical region we observed in human male individuals was in agreement with previous findings (Pelletier et al., 1987; Mouri et al., 1993), except that we have not detected labeled cell bodies in the periventricular region, unlike one previous study (Pelletier et al., 1987). In different species, the axons arising from MCH neurons abundantly innervate the hypothalamic preoptic and periventricular areas, the medial septum/diagonal band of Broca, the lateral septum, the LHA, the arcuate/premamillary area and the eminentia mediana (Bittencourt et al., 1992, 1998; Tillet et al., 1996; Elias et al., 1998; Chiocchio et al., 2001; Gallardo et al., 2004; Williamson-Hughes et al., 2005). Here, we found that the MCH-IR projections innervated the preoptic and infundibular regions where large subsets of GnRH neurons are located in humans (Dudás et al., 2000; Hrabovszky and Liposits, 2013). In line with earlier observations in laboratory rodents (Williamson-Hughes et al., 2005; Ward et al., 2009; Wu et al., 2009), our present immunohistochemical experiments provided evidence for direct axo-somatic and axo-dendritic connections between human MCH and GnRH neurons. We note that this innervation was about 50% less heavy than the ORX-ergic input what we studied in histological specimen processed in parallel. This difference can have biological (more ORX than MCH fibers innervating GnRH cells) as well as technical (e.g., better ORX *vs.* MCH antibody quality) explanations.

Functional studies of rodents revealed that GnRH/LH release can be influenced by exogenous MCH. As also noted above for ORXs, the effect of MCH is bimodal, depending on estrogen levels; MCH stimulates and suppresses GnRH/LH secretion under conditions of high (Gonzalez et al., 1997; Murray et al., 2000a,b; Chiocchio et al., 2001) and low (Tsukamura et al., 2000) estrogens, respectively, *in vitro*, as well as after intracerebroventricular administration of the peptide. Estrogens regulate MCH and MCHR1 protein expression negatively, as shown in ovariectomized rats treated with estradiol and in intact females during proestrus (Santollo and Eckel, 2013). The reduced MCH and MCHR1 protein detected with immunohistochemistry and in immunoblots, respectively, do not seem to result from the direct effects of estradiol on MCH neurons, since estradiol-treatment does not change MCH or MCHR1 mRNA expression in cultured hypothalamic neurons (Santollo and Eckel, 2013). Furthermore, MCH neurons do not express the alpha estrogen receptor isoform (Muschamp and Hull, 2007; Santollo and Eckel, 2013).

MCH acts via the G-protein coupled receptor MCHR1 (SLC-1/GPR24; Bächner et al., 1999; Chambers et al., 1999; Lembo et al., 1999; Saito et al., 1999; Shimomura et al., 1999). MCHR1 can activate multiple signaling pathways via coupling to different intracellular effector proteins G_i_, G_o_ and G_q_ (Hawes et al., 2000). While MCH can cause increased cytoplasmic calcium and excitation of MCH receptor expressing non-neuronal cells (Gao and van den Pol, 2002), most studies found that MCH inhibits neuronal firing (Pissios et al., 2006). Accordingly, MCH attenuates the calcium currents and inhibits neuronal activity in lateral hypothalamic neurons due to the G_i_ coupling of MCHR1 (Gao and van den Pol, 2001, 2002). Similarly, MCH was also found to inhibit GnRH neuronal activity in mice and interrupt the persistent excitatory effect of kisspeptins on GnRH cells (Wu et al., 2009).

Our study has not addressed the presence of MCH receptors in GnRH cells. Of note, a second MCH receptor form (MCHR2) with high-affinity MCH binding has also been identified in human (An et al., 2001; Sailer et al., 2001; Wang et al., 2001). Unlike MCHR1, MCHR2 couples only to the G_q_ protein, indicating its involvement in stimulatory neurotransmission. Therefore, the clarification of the receptor subtype/s in human GnRH cells may have important functional implications.

Overall, anatomical evidence in this study suggests that MCH has a direct effect on human GnRH neurons. Similarly to ORXs, the presence of MCH in neuronal afferents to human GnRH cells may play an important role in linking energy balance and other LHA-related functions to reproduction.

### The Dominance of Dendritic Over Somatic Inputs to GnRH Neurons

Results of early electron microscopic experiments in rats and monkeys established that the cell body of GnRH neurons receives only very few (2–12) synaptic inputs (Witkin and Silverman, 1985; Witkin et al., 1991, 1995). More recent studies of biocytin-filled mouse GnRH neurons revealed lengthy GnRH dendrites with numerous dendritic spines which received excitatory synaptic inputs quite abundantly (Campbell et al., 2005). These newer observations suggest that GnRH neurons are more heavily innervated than thought previously (Witkin and Silverman, 1985; Witkin et al., 1991, 1995) and the majority of inputs may target the dendritic compartment. The preferential innervation of the dendritic *vs*. the somatic compartment may also be the case in the human. Results of recent studies from our laboratory have established that the human GnRH dendrite receives very dense GABAergic and glutamatergic innervation (Hrabovszky et al., 2012a). A recently proposed anatomical model of the neurosecretory GnRH cell suggests that the hypophysiotropic projections of GnRH neurons exhibit several dendritic rather than axonal properties and receive abundant synaptic innervation (Herde et al., 2013). While this observation has not been extended to the human yet, the hypophysiotropic projections of human GnRH neurons are often even much longer than in rodents, and may offer large membrane surfaces to receive synaptic inputs. In addition to dendrites, hypophysiotropic GnRH axon projections in the vicinity of the infundibular stalk which approach and surround the portal vasculature may serve for additional sites of neuronal interactions (Hrabovszky and Liposits, 2013; Borsay et al., 2014). Of note, we have recently shown that the lengthy hypophysiotropic GnRH projections intermingle with the plexuses of kisspeptin, neurokinin B and substance P neurons and establish occasional axo-axonal appositions with these systems (Borsay et al., 2014). In the present study, we observed the majority (77–81%) of ORX/GnRH and MCH/GnRH appositions on the dendritic compartment of GnRH neurons. While the axo-dendritic inputs seem to represent the dominant form of communication between human MCH and GnRH neurons, significant axo-axonal interactions did not seem to exist in the infundibular stalk and postinfundibular eminence which contained relatively few ORX and MCH fibers in our study. In contrast, the rat eminentia mediana was reported to contain MCH fibers both in its internal and external layers (Bittencourt et al., 1992; Chiocchio et al., 2001) and proposed to represent an important site where MCH may stimulate GnRH secretion in this species (Chiocchio et al., 2001).

In summary, our study provides morphological evidence for the afferent regulation of human GnRH neurons by lateral hypothalamic ORX and MCH neurons.

## Author Contributions

KS, VK, ZS, CM, OS, AH, BÁB, LH, ZL and EH conceived and designed the experiments and wrote the paper. BÁB and LH contributed essential research material.

## Conflict of Interest Statement

The authors declare that the research was conducted in the absence of any commercial or financial relationships that could be construed as a potential conflict of interest.
